# Immigration, Discrimination, and Trust: A Simply Complex Relationship

**DOI:** 10.3389/fsoc.2019.00032

**Published:** 2019-05-17

**Authors:** Rima Wilkes, Cary Wu

**Affiliations:** Department of Sociology, University of British Columbia, Vancouver, QC, Canada

**Keywords:** immigration, trust (social and political), mediation analysis, discrimination, race

## Abstract

Many immigrants experience discrimination. In this paper we consider how discrimination affects their trust. We make a theoretical case for a formal mediation approach to studying the immigration, discrimination, and trust relationship. This approach shifts attention to the basic fact that the overall *levels* of discrimination experienced by different immigrant and native-born groups are not the same. We also build on previous empirical research by considering multiple forms of discrimination, multiple types of trust and multiple immigrant/native-born groups. Drawing on the 2013 Canadian General Social Survey data (*N* = 27,695) we analyze differences in three kinds of trust (generalized trust, trust in specific others, and political trust), and the role of perceived discrimination (ethnic, racial, any), between five immigrant-native groups (Canadian-born whites, Canadian-born people of color, foreign-born whites, foreign-born people of color, and Indigenous people). We find that perceived discrimination is more relevant to general trust and trust in specific others than to political trust. We also find that perceived discrimination explains more of the trust gap between racialized immigrants and the native-born than the gap between non-racialized immigrants and the native-born. The results illustrate that what appears to be a simple relationship is far more complex when attempting to explain group differences.

## Introduction

Although there are a few exceptions, many immigrants as well as other ethnic and/or minority group members tend to trust less in generalized others (Smith, 2010; Ziller, 2017). Nor is this surprising given the discrimination that minority group must often endure. The trust gap extends to immigrants and non-immigrant groups in a variety of immigrant receiving societies in the European context (Kotzian, 2011; Mewes, 2014) including Denmark (Bjørnskov, 2008) and the Netherlands (De Vroome et al., 2013). Trust gaps have also been documented in the North American context including in Canada and the United States (Chávez et al., 2006; Hwang, 2017). Conversely, in the case of particularized social trust in family, friends and relatives, immigrants tend to trust their own group members more than out-group members (Uslaner, 2017). Finally, when it comes to political trust, the relationship appears to be the opposite—immigrants tend to trust government more than the native-born or is at the very least mixed (Bilodeau and Nevitte, 2003).

While many studies have explored how discrimination might matter for immigrants' trust (e.g., Dinesen, 2010, 2012; Dinesen and Hooghe, 2010), no study has been able to delineate whether discrimination against immigrants occurs as a result of immigrantstatus or because of race (via the process of racialization). A key way to think about this relationship is that migrants in a new society have effectively changed their relative position (Wilkes and Wu, 2018)—many becoming not only “migrants” but also “racialized minorities.” Consider, for example, migrants from China. When they are in China the vast majority (if they are Han Chinese) will be in the ethnic majority group. Upon arrival in any new destination they will likely be in the minority group. And, it is very likely that, in this new social position, they will be subjected to discrimination. For example, in their study of the experience of Chinese immigrants in the United States Qin et al. (2008) provide the example of student from Hong Kong who says about the bullying that he experiences “In Hong Kong, no one treats me like that…They are not targeting one individual student, they target the entire group of Chinese students.” This is not an isolated example.

In this paper we consider how discrimination mediates the relationship between nativity and different kinds of trust. We argue that, a focus on *who* discrimination matters for, and for what kind of trust, can be used to explicate the meaning of immigrant-native gaps in trust. In the above case it appears that the student was targeted due to being an immigrant. But, in the U.S. context the student is also a racialized minority. Native-born racialized minorities also trust less (Smith, 2010; Wilkes, 2011). Both groups often have higher political trust. Therefore, we ask, are these trust gaps and the impact of discrimination reflective of the nature of being an immigrant or are they reflective of being a racialized (minority)? To what extent does the answer to this question in turn, depend on the type of trust? An answer to these questions requires disentangling the effects of discrimination based on nativity from the effects of discrimination based on racialization.

We do so by considering how different categories of nativity, race and discrimination operate on trust within the Canadian context^1^. As a high-immigration and high trust society, Canada provides an ideal case with which to think about these relationships. Although Canada is a high trust society globally, there are still group differences in trust (Soroka et al., 2006). Similarly, while Canada also has an international reputation for diversity and a policy of official multiculturalism enacted in 1988 it has not been immune to problems of ethnic and racial discrimination. The data for this study comprise the 2013 Identity Cycle of the Canadian General Social Survey. We use this dataset to test whether the potential mediating effect of discrimination on the immigrant gap in trust is about race or nativity.

## Immigration and Trust

Trust is invisible. While we can see the manifestation of many social science topics such as protests, homicides, births, and urban disorder, this is not the case with trust. Nevertheless, trust is essential to our very existence as social beings, similar to the role of oxygen for our biological survival. Society as we know it is not possible without trust. Trust correlates with important individual-level benefits including increased life satisfaction, health, and happiness (Helliwell et al., 2014).

As such, trust is a positive topic. Unlike issues such as terrorism, environmental disasters, genocide, and poverty, trust doesn't appear to be an urgent “problem.” And yet, as the recent explosion of psychological research on happiness illustrates, it is also the case that while negative topics such as depression, anxiety and suicide once predominated positive topics are now widely accepted as being as important (see e.g., Diener discussion in Belic, 2011). Trust is similar. Even though it is not an obvious problem *per se* it is at the same time vital for our well-being. For these reasons trust has become one of the most significant areas of social science inquiry (Uslaner and Brown, 2005).

Trust is “a generalized expectancy held by an individual or a group that the word, promise, verbal, or written statement of another individual or group can be relied on” (Rotter, 1971: 44). It appears to be a simple concept but has been the subject of considerable debate. Some scholars say that trust is a form of “social credit” or “encapsulated interest” in which an individual does something for another with a view to a future return (Coleman, 1988; Hardin, 1999). Others say that it is less instrumental and more about whether the object of trust is concerned with one's general interest and well-being.

Based on the object or targets, trust can take several forms including social (generalized, specific) and political-institutional. *Generalized trust*– typically indicated by the question “most people can be trusted” refers to a generalized and unknown other. This form of trust has been shown to positively impact a host of other desirable outcomes including social cohesion (Putnam, 1993, 1995, 2001) “well-being,” and “governance” (Uslaner, 2017; p. 1). *Specific trust* in targeted groups such as family, friends, or relatives, or even racial and ethnic groups is integral for group cohesion and inter-group relations (Yuki et al., 2005). *Political trust* is needed for effective government functioning (Citrin, 1974; Easton, 1975; Wu and Wilkes, 2018a). Government cannot make effective policy or difficult decisions if its citizens do not trust it to do the right thing.

It has been well-established that on average immigrants tend to trust generalized others less than the native born (see Bilodeau and Nevitte, 2003; Kazemipur, 2006; Nakhaie, 2008; Stolle et al., 2008; Doerschler and Jackson, 2012; Hwang, 2013; Nakhaie and de Lint, 2013). This finding holds in Canada (Baer et al., 1993; Hwang, 2013), Europe (Kotzian, 2011; Mewes, 2014) and for some groups in the United States (Uslaner, 2008). In the case of particularized social trust, in-group members tend to trust their own group members more than out-group members (Uslaner, 2017). On the other hand in the case of political trust, whereas many racialized native-born groups such as Black Americans and Indigenous peoples generally have lower political trust (Avery, 2006, 2010; Wilkes, 2014, 2015; Hwang, 2017), some immigrant groups tend to have higher political trust (Nevitte and Bilodeau, 2003; Bilodeau and White, 2016). These trust gaps matter not only for the individuals themselves but also for larger societal cohesion.

Several studies attribute the lower generalized trust of immigrant groups to the fact that they came from societies that engender distrust (Uslaner, 2008; Dinesen, 2012, 2013; De Vroome et al., 2013; Ziller, 2014). This argument has been tested by looking at whether trust levels are different between migrants (in a new society) and natives (in new society) as well as what the mean trust level is in the point of origin. However, as the above examples illustrate, there is still fuzziness around whether trust gaps are reflective of differences in the experience of nativity or differences based on racialization (or both) and if so how this might be tied to discrimination in the new society.

## Discrimination as a Mediator

Discrimination refers to “inappropriate and potentially unfair treatment of individuals due to group membership.” (Dovidio et al., 2008: p. 8; see also Allport, 1979). While discrimination is a behavior or experience, its roots are prejudicial (that is negative) attitudes about a given individual based on stereotypical attitudes about the group that an individual is perceived to belong to Pettigrew (1998). As a number of scholars who conduct experimental research show, discrimination does not necessarily occur at a conscious level (Foschi, 2000; Ridgeway, 2001). Clearly while discrimination can occur based on many perceived characteristics—age, gender, appearance etc.—of interest here is racial and ethnic discrimination. As Quillian (2006: p. 302) notes, “discrimination is the difference between the treatment that a target group actually receives and the treatment they would receive if they were not members of the target group but were otherwise the same.”

Discrimination is likely to be a particularly salient predictor of trust because, rather than being a characteristic that, to some extent one might come to terms with or even change, it is an external set of events and experiences that shapes one's ability to successfully navigate life within a larger society. Furthermore, because of these experiences, and their day-to-day unpredictability, individuals can never be sure where or when these experiences will occur again. Individuals who have experienced discrimination must always be on their guard, and cannot therefore, afford to trust. This is why “minorities who feel discriminated against will be less sanguine about their prospects for sharing in society's bounty.” (Rothstein and Uslaner, 2005: p. 51). Kumlin and Rothstein (2007), for example, make the case that if individuals experience discrimination in one avenue, such as the political sphere, this will also spill over and affect other forms of trust, including trust in others.

There are a few studies that have considered the relationship between discrimination and trust and that have done so for specific immigrant groups. Liebkind and Lahti (2000), for example, find that discrimination affects confidence in institutions for six of seven immigrant groups in Finland. Kääriäinen and Niemi (2014) analyzed the association between the experience of discrimination and trust in the police for Russian and Somali minorities in Finland but only found a relationship for Somalis, a finding that they attribute (though do not test directly) to racial discrimination. Schildkraut (2005) finds that perceptions of individual-level discrimination lowers Latinos' trust in the U.S. government. In contrast, Dinesen (2010) finds that early experiences of discrimination does not affect generalized trust among Danish immigrants.

**Figure 1:**
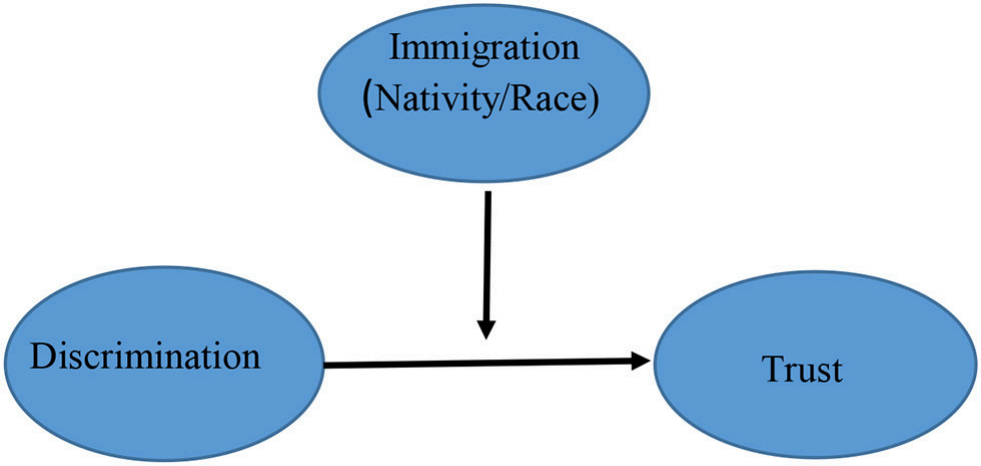
Moderating relationship.

**Figure 2:**
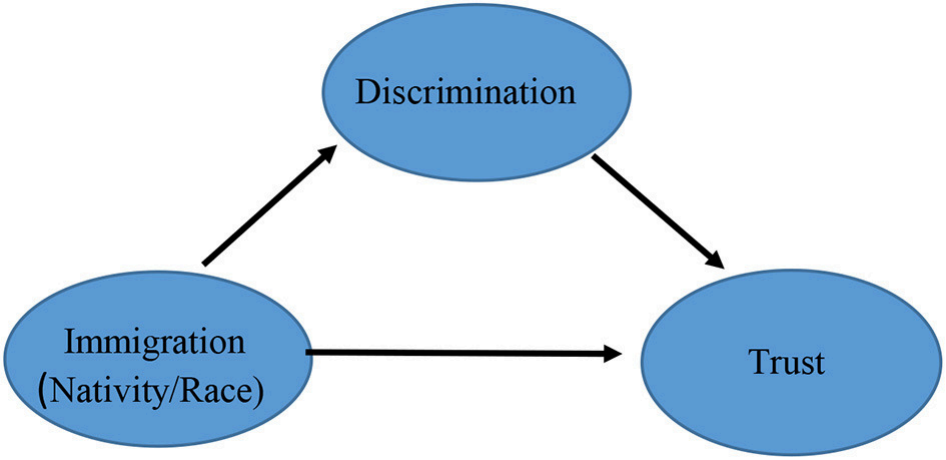
Mediating relationship.

Still, the predominant approach in these studies is to consider the effect of discrimination on trust across immigrant groups or to consider the relationship between discrimination and trust within immigrant groups (moderation). Figure 1 shows the standard moderating approach to the immigration-discrimination trust relationship. Essentially this approach is testing whether the effect of discrimination on trust is variable—that is whether the trust of some immigrant groups is more sensitive to discrimination. However, this approach cannot explain the extent to which discrimination accounts for the gap in trust between immigrants and the native-born. That is the fact that the *levels* of discrimination are higher (or potentially so) for immigrant and/or racialized groups also needs to be taken into account and done so as more than a control. This is because conceptually what matters is the fact that immigrants typically experience more discrimination than the native-born. While a small minority may have experienced discrimination in their place of origin discrimination is an experience that is a function of location within the new host society (see also Dinesen, 2012, 2013 on the move from low to high trust societies). This latter scenario suggests a mediating rather than a moderating relationship, and therefore is an explanation where the emphasis is on why, rather than how, groups differ (Reskin, 2003). Figure 2, above, illustrates this mediating relationship where the effect of immigrant status is explained by discrimination and the focus is on *gaps* in trust. In both cases we include nativity and race as subcomponents of the concept of immigration.

Only one study to date (Röder and Mühlau, 2012) has attempted to think about the relationship between discrimination and trust in this way and it does not actually measure discrimination. Röder and Mühlau (2012) test whether discrimination can account for differences in confidence in public institutions between first and second-generation migrants and native born in 26 European countries between 2002 and 2006 and find little effect. However, all the measures they use to indicate discrimination—whether the respondent is an ethnic minority, practices a non-Christian religion, speaks a different language or is a member of a group that experiences discrimination—actually indicates discrimination—are, arguably, indicators of different aspects of ethnicity rather than indicators of discrimination. The few studies that have used a mediation approach to considering explain ethnic differences in trust (e.g., see De Vroome et al., 2013; Wilkes and Wu, 2018) do not have a measure of discrimination and do not use formal mediation analysis. Similarly, while Douds and Wu (2018) include models that look at how discrimination mediates the Black-White and Hispanic-White gaps in trust in Louisiana, they do not use a formal test of mediation. The strength of the mediation framework is that not only does it allow us to assess whether discrimination explains some of the impact of immigrant status on trust but also, as we highlight below, the relative importance of discrimination in explaining trust for different immigrant and native born and racialized groups.

To test this relationship empirically requires datasets that contain sufficient numbers of immigrants and racialized. The problem is that in most datasets the number of respondents who identify as immigrants and/or minority is small (except see e.g., Uslaner, 2011; Dinesen, 2012; De Vroome et al., 2013). A country-specific dataset, might for example, only contain a thousand respondents and will therefore only have responses from a limited number of immigrants and or racialized minorities. Helliwell et al. (2014) have a sample of over 6,000 immigrants but because this sample is derived from 127 countries there is only an average of 47 immigrants per country. Doerschler and Jackson (2012) compare 96 Muslims in Germany to over 3,000 non-Muslims. Nannestad's et al. (2014) comparison trust of various ethnic groups in Denmark includes 276 Turks, 267 Pakistanis, 115 Bosnians and 64 Ex-Yugloslavians. This then precludes a detailed analysis of heterogeneity within minority populations (except see De Vroome et al., 2013). The other issue is that, while most trust datasets such as the World Values or European Social Surveys contain indicators of social capital and socio-economic status, there are typically no direct measures of discrimination.

We do so here using data from the 2013 Canadian General Social Survey Cycle 27, Social Identity and Giving Volunteering and Participating collected by Statistics Canada. As a high-immigration and high trust society, Canada provides an ideal case with which to think about this relationship. While Canada has an international reputation for diversity and a policy of official multiculturalism it has not been immune to problems of discrimination. Of further relevance is that, as of 2016, over one fifth of Canada's population is foreign-born (Statistics Canada, 2016). This, in combination with the very large sample size of the CGSS (*N* = 27,695), means that there are over 9,000 immigrants (and over 6,000 people of color).

With these distinctions in mind it can be expected that discrimination (partially) mediates the immigrant-native gap in trust within the Canadian context. That is, immigrant minority status is associated with increased discrimination, which, in turn, decreases trust—immigrant status has an indirect effect on trust via discrimination. More specifically, if this relationship only exists for the race-based groups then the effect of nativity on trust is about being discriminated as a racialized minority. Conversely, if this relationship between immigration, discrimination, and trust only exists for the nativity-based groups then the effect on trust is about being discriminated as an immigrant minority, possibly due to some other factor such as language or social stereotyping about place of origin. Assuming four possible groups for comparison (Canadian-born whites, Canadian-born racialized minorities, foreign-born whites, foreign-born racialized minorities) there are then three possible hypotheses.

H1: Discrimination will mediate the difference in trust between Canadian-born whites and all others (Canadian-born people of color, foreign-born whites, and foreign-born people of color). This would indicate that the impact of discrimination on trust mediates the effects of both nativity and racialization.

H2: Discrimination will mediate the difference in trust between the Canadian–born (white and people of color) and the foreign-born (white and people of color). This would indicate that the impact of discrimination on trust only mediates the effects of nativity.

H3: Discrimination will mediate the difference in trust between whites (Canadian-born and foreign-born) and people of color (Canadian-born and foreign-born). This would indicate that the impact of discrimination on trust only mediates the effects of racialization.

This said these hypotheses focus primarily on whether gaps in trust exist across various nativity and racialized groups. As currently stated these hypotheses generalize across all three types of trust. Which of these hypotheses is the case may also depend on the type of trust. Thus, for each type of trust there are three possible hypotheses to be tested.

## Data

The GSS Social Identity model is designed to “understand how social integration is being built among people living in a modern, diverse society with multiple ethnicities and backgrounds” (Statistics Canada, 2013). Statistics Canada further states that “questions on social networks and norms of trust will examine the social patterns that hold society together.” The dataset contains multiple measures of discrimination. Most datasets either contain too few minorities and/or contain no direct measures of discrimination.

### Immigrant, Native, and/or White/People of Color and Indigenous Groups

We identify immigrants and native-born using the place of birth (brthcan), Place of birth asks whether the respondent was born in or outside Canada. While we do not have group-specific identifiers we do have a yes/no visible minority question (vismin)^2^, and Aboriginality (AMB_01) variables. We have replaced the terms visible—non-visible minority with the terms people of color white throughout this paper. We do not use visible minority because in the Canadian context this is a misnomer—for example as of 2018 in Vancouver and some of the surrounding areas the visible minority is white.

We also use Indigenous rather than Aboriginal because this is the more widely used term in the contemporary Canadian context. Further is that while Indigenous people are clearly the original “native-born,” they cannot be placed into the same category as non-Indigenous native-born. This is because Indigenous people's “identity exists in an uneasy balance between concepts of generic “Indianness” as a racial identity and of specific “tribal” identity as Indigenous *nationhood*.” (Lawrence, 2003: p. 4; see also Cardinal, 1999; Christie, 2005). This, in conjunction with the fact that the nature of the discrimination that Indigenous peoples encounter may be qualitatively different, necessitates their inclusion as a separate group.

We combined these questions to identify Canadian-born whites, Canadian-born people of color, foreign-born whites, foreign-born people of color and Indigenous people^3^, Note that we replace the term native-born with Canadian-born hereafter. Indigenous people include all individuals who identify as Aboriginal—First Nations, Inuit, and Métis.

### Dependent Variables: Generalized, Specific, and Political Trust

We consider three different types of trust –two kinds of social trust—generalized trust (in unknown others), and trust in more specific others- as well as political trust. Generalized trust is measured using the Trust people in general (TIP_10) question which asks respondents whether “generally speaking, would you say that most people can be trusted or that you cannot be too careful in dealing with people?” This is a binary measure with its two outcomes “Most people can be trusted” and “You cannot be too careful in dealing with people.” Specific social trust is measured with an additive index of Trust in people in the neighborhood (TIP_15), Trust in people who speak a different language (TIP_22), and Trust in strangers (TIP_25). All three were coded on a 1–5 scale with 1 denoting “Cannot be trusted at all” and 5 denoting “Can be trusted a lot.” A factor analysis of a larger list of questions on specific others indicated that these three were congruent (factor loadings are 0.77, 0.74, and 0.64) and we therefore included them in an index that we then re-scaled from 1 to 5 by dividing by three. We measure political trust using a similar index of three variables denoting how much confidence the respondent has in the police (CII_Q1), the justice system and the courts (CII_Q15), and the Federal Parliament (CII_Q40). All three were coded on a 1–5 scale with 1 denoting “No confidence at all” and 5 denoting “A great deal of confidence.” These particular objects of trust have been widely used in trust studies and load on a single factor (factor loadings are 0.65, 0.77, and 0.65) (see also Wu and Wilkes, 2018b).

### Mediators: Perceived Discrimination

Discrimination (perceived) is measured with three questions indicating “whether the respondents experienced discrimination” on the basis of ethnicity (DIS_15), race (DIS_20), or any discrimination at all in the past 5 years (discrim). Therefore, this was a series of outcomes preceded by the experienced discrimination statement. In the latter case this could include perceived discrimination on the basis of ethnicity, race as well as gender, age or some other characteristic. All are binary measures with 1 denoting yes and 0 denoting no. Because a factor analysis showed that, with the exception of the first two, these do not load on the same component or within all groups, we do not include them in an index.

### Control Variables: Socio-Economic Status, Social Capital, and Demographics ^4^

Socio-economic status is denoted by education (DH1GED) and work status (MAR_110). Education is a four-category variable with 1 indicating less than high school, 2 graduated from high school, 3 post-secondary diploma and 4 university degree. The work variable originally had 10 categories and because there were small numbers in many of these groups we recoded this measure to denote four groups—working full or part time, student, retired, and other. We also ran all analyses using the household income (incmhsd) variable—the results are similar- but do not retain it as it is not our key focal measure and because, at 22% its rate missingness was too high (see footnote 8).

Social capital, an important control in any study of trust, is measured with volunteering (VCG_300) and number of friends (SCF_100C). The volunteer measure is a yes/no indicator of whether the respondent volunteered in the last 12 months. The number of friends was an open-ended question asking about the number of close friends. To eliminate skew at the top end of this measure we recoded all responses above 11 in the 11 category. Though not a social capital measure *per se*, we also control for political interest (REP_05) which asks respondents about their interest in politics from “very interested” to “not interested at all.” We recoded this variable so that the not interested categories was at the low end of the scale and the very interested was at the high end of the scale.

Demographics include age (AGEGR10), sex (sex), and marital status (marstat). Age is measures on a 7-point scale denoting from low to high the following age groups: 15–24, 25–34, 35–44, 45–54, 55–64, 65–74, and 75 and over. Sex is a binary with one denoting the effect of being female. Marital status originally had six categories that we recoded into a binary measure denoting married vs. all others. We also include urban residence (LUC_RST) in large part because this may have a unique distribution across groups within the Canadian context where many Indigenous peoples live in rural areas and on reserve. This measure is coded as 1 if the respondent lived in a larger urban population centers (CMA/CA) vs. 0 if they resided in a rural areas/small population centres (and also Prince Edward Island which is coded as a separate category and was recoded as 0).

## Methods

In addition to general descriptive and bivariate analysis, we conducted multivariate analyses with a view to ascertaining the extent to which the discrimination variables (M—mediator) mediate the effect of the immigration measures (X—independent variable) on trust (Y—dependent variable). As Preacher (2015: p. 846) notes, because it depends on a host of factors including theory, study design, the data, and the sample “there is no universally correct approach” to mediation. Until relatively recently, the standard formal approach to mediation analysis has been Baron and Kenny's (1986) 3-step method where (1) X was regressed on Y; (2) M was regressed on Y; and finally, (3) M was regressed on X (see e.g., Carpiano and Hystad, 2011)^5^. If all three models show a significant effect then this provides evidence of a mediating relationship, the significance of which is confirmed with a Sobel (1982) test. Although widely-used (Baron and Kenny have been cited over 24,000 times), this approach requires a model with a single rather than multiple mediators, a single rather than multiple independent variables, continuous measures, and a dataset that has a large sample size.

While we do have a large sample size we also have multiple mediators (three binary measures of perceived discrimination—ethnic, racial, and any discrimination), a multi-category set of independent variables, and three outcome measures, one of which—generalized trust—is binary rather than continuous. We use the formal Kohler et al. (2011) (KHB) method which was developed to compared “the estimated coefficients of two nested probability models” (420). There are two reasons why we use this particular method. First, in the case of binary outcomes such as the generalized trust measure, the KHB method addresses the issue of rescaling (e.g., see Mood, 2010; Christensen and Carpiano, 2014; Yang and Park, 2015). Second, the KHB method can be used with multi-category independent variables as well as multiple mediators (Kohler et al., 2011). All multivariate analyses are weighted by the individual WGHT_PER variable^6^.

## Findings

Table 1 provides the descriptive statistics for the dependent (Y) trust measures, mediating (M) perceived discrimination measures, and control variables across the five independent (X) nativity groups. In terms of trust, irrespective of its type, there are clear differences across groups^7^. Generalized trust is highest among foreign-born Whites. People of color, irrespective of where they are born, have equal levels of generalized trust and it is lowest among Indigenous people. In terms of trust in specific others, it is highest for whites, irrespective of place of birth, followed by Indigenous and lowest for persons of color. Finally, turning to political trust the results show that it is highest among foreign-born persons of color and whites. Levels are lower for the Canadian born group but are the same based on visible minority status. Political trust levels are lowest for Indigenous respondents.

**Table 1:** Mean trust levels, by nativity.

**Nativity status**	**Canadian-born**		**Foreign-born**		**Indigenous**
	**White**	**Person of Color**	**White**	**Person of Color**	
**Dependent variables (Y)**					
Generalized social trust (0.1) (1 = trusting)	0.54	0.49	0.6	0.49	0.45
Trust in specific others (1–5) (low to high)	3.29	2.93	3.32	2.94	3.02
Political trust (1–5) (low to high)	3.51	3.52	3.72	4.01	3.36
**Independent variables (X)**					
Ethno-racial group	61	2	14	20	3
**Mediators (M)**					
Ethnic discrimination (0.1) (% yes)	4.63	32.24	12.07	29.12	22.62
Racial discrimination (0.1) (% yes)	3.82	33.72	5	28.82	19.57
Any discrimination past 5 years (0.1) (% yes)	25.99	49.42	28.48	40.8	44.61
**Controls**					
Age (mean 1–10 scale)	4.19	2.14	4.29	3.03	3.54
Female (0.1) (% yes)	55	53	52	52	59
Married (0.1) (% yes)	58.53	32.94	64.85	61.61	54.8
Rural (0.1) (% yes)	23	3	12	2	33
Less than high school (%)	17.53	22.83	10.03	11.89	24.58
Graduated high school (%)	27.37	31.13	21.79	24.41	31.24
Some post-secondary (%)	33.16	19.43	32.44	24.32	32.42
University (%)	21.94	26.6	35.74	39.38	11.76
Employed (%)	55.03	49.3	55.12	62.86	55.13
School (%)	6.79	34.11	7.59	18.22	10.1
Retired (%)	26.11	7.24	24.94	5.95	14.57
Other (%)	12.07	9.35	12.35	12.97	20.2
Number of friends	5.1	5.6	5.2	4.9	4.9
Volunteer (% yes)	36.5	44.2	36.2	34.1	38.7
Political interest	2.8	2.5	2.8	2.5	2.6
*N*	17,020	534	3,877	5,600	835

*All means and percentages are calculated using Statistics Canada bootstrap weighting*.

The results also show that, not surprisingly, there are stark differences in the rates of perceived discrimination experienced by the members of different groups. Canadian-born people of color experience (or are the most likely to report such experiences) the highest rates of all forms of perceived discrimination (except physical) across the board. About a third of the members of this group report ethnic and racial discrimination and almost half report some form of discrimination in the previous 5 years. Foreign-born people of color and Indigenous people also report high rates of ethnic and racial discrimination^8^. Finally, as might be expected, we see that the rates of ethnic and racial and overall discrimination are much lower for the two White populations. Still, at least one quarter of both groups report experiencing some form of discrimination in the previous 5 years^9^. These higher rates of discrimination among the racialized minority groups show that the more frequent experience of discrimination is a likely explanation for why minorities could have lower trust than majority group members.

The distribution of the control variables is considerably different across the five groups, indicating that it is important that these be included in any model of generalized trust. For example, the foreign-born population has higher rates of university completion than the Canadian-born. This group (of color) also has higher rates of employment. The foreign-born person of color group is most likely to be employed and Canadian-born person of color the least. The rates are similar across the other three groups. The distribution of social capital does vary across groups, though not as markedly as it does for some of the other categories. The white population (native and foreign-born) is considerably older than the person of color population and the Indigenous population. The Canadian-born person of color population is also less likely to be married than any of the other groups. Also persons of color (irrespective of place of birth) are far less likely to live in rural areas than either white populations or Indigenous people.

Table 2 provides the results of the mediation analysis of the logistic regression analysis of generalized trust including controls for demographics, SES and social capital. The first column shows the log odds on trust of a given pathway and the second and third columns show, respectively, whether this pathway is statistically significant and the robust standard error. For each group we provide the total effect—which refers to the gap in trust between that particular group and Canadian-born whites. The next two rows split that effect into the portion of the total effect that is direct and the portion of the total effect that is mediated via the perceived discrimination variables. The latter two add up to the total effect. The fourth column shows the percentage of the total effect accounted for by the mediation pathway. This percentage should be interpreted cautiously insofar as a greater percentage does not de facto imply a greater overall effect—a larger percentage may be explaining a very small effect. The fifth column shows the percentage of that total effect attributed to each mediating variable in the model.

**Table 2:** KHB mediation analyses of extent to which discrimination mediates effect of nativity status on generalized trust.

			**Robust**	**Overall**
	**Estimate**		**SE**	**Mediation %**
**VS. CANADIAN-BORN WHITE**				
**Canadian-born person of color**				
Total effect	−0.317	^*^	0.127	
Direct effect	−0.144		0.128	
Mediating effect	−0.173	^***^	0.035	54.66
**Foreign-born white**				
Total effect	0.121	^*^	0.057	
Direct effect	0.140	^*^	0.057	
Mediating effect	−0.019		0.024	−15.94
**Foreign-born person of color**				
Total effect	−0.339	^***^	0.060	
Direct effect	−0.210	^***^	0.062	
Mediating effect	−0.129	^***^	0.031	38.11
**Indigenous**				
Total effect	−0.226	^*^	0.102	
Direct effect	−0.116		0.103	
Mediating effect	−0.110	^***^	0.027	48.68

* p < 0.05; ^**^ p < 0.01;

*** *p < 0.001*.

The results in Table 2 show that perceived discrimination is the primary cause of the gap in generalized trust between Canadian-born people of color and Canadian-born whites (discrimination explains 54.6 % of the gap). The total effect or gap between these groups is −0.317, a gap that becomes much smaller once the mediating effect of perceived discrimination −0.173 is taken into account—or, as column four shows- almost 54.6% of the effect (e.g., partial mediation). This particular mediating effect operates primarily through ethnic discrimination (42%) and to a lesser extent through racial and any discrimination (about 28%, respectively). In contrast, the results show that perceived discrimination does not mediate the gap in trust between foreign-born and Canadian-born whites (a gap that favors foreign-born whites). The fact that the overall percentage explained by discrimination is negative indicates that, if anything, discrimination is suppressing other factors. For foreign-born people of color as well as for Indigenous people the pattern is similar to Canadian-born people of color. There is lower generalized trust and there is partial mediation of the gap via perceived discrimination. In this instance, discrimination explains 38 and 48% of the gap, respectively.

Table 3 provides the results of the analysis of the OLS regression analysis of trust in specific others. The total effects show a similar pattern to generalized trust. There is a negative gap in trust between Canadian-born people of color, foreign-born people of color and Indigenous people indicating that the members of the former groups have lower trust on average than Canadian-born whites (−0.171, −0.310, and −0.191, respectively). Perceived discrimination partially mediates these gaps, and, as with generalized trust, the group most explained by discrimination is Canadian-born people of color (42%).

**Table 3:** KHB mediation analyses of extent to which discrimination mediates effect of nativity status on trust in specific others.

			**Robust**	**Overall**
	**Estimate**		**SE**	**Mediation %**
**VS. CANADIAN-BORN WHITE**				
**Canadian-born person of color**				
Total effect	−0.171	^***^	0.043	
Direct effect	−0.099	^*^	0.044	
Mediating effect	−0.073	^***^	0.014	42.39
**Foreign-born white**				
Total effect	−0.005		0.023	
Direct effect	0.001		0.023	
Mediating effect	−0.007		0.011	125.48
**Foreign-born person of color**				
Total effect	−0.310	^***^	0.022	
Direct effect	−0.255	^***^	0.023	
Mediating effect	−0.055	^***^	0.013	17.8
**Indigenous**				
Total effect	−0.191	^***^	0.041	
Direct effect	−0.142	^***^	0.041	
Mediating effect	−0.049	^***^	0.011	25.57

* p < 0.05; ^**^p < 0.01;

*** *p < 0.001*.

Table 4 provides the results of the analysis of the OLS regression analysis of political trust. For Canadian-born people of color it appears that there is very little overall gap in political trust with Canadian-born whites. However because the direct effect is positive (0.075) and the mediating effect via discrimination is negative (−0.108) this is a case of competitive mediation, that is, a pattern where the mediated and direct effect are approximately the same size but operate in different directions (see Zhao et al., 2010). This also explains why the overall mediation percentage is so large. There is no mediating effect of discrimination for foreign-born whites but it does depress the political trust of foreign-born-persons of color (−0.081). Importantly, is that the total effect is positive for both foreign-born groups indicating that political trust is higher than that of the Canadian-born (see also Bilodeau and White, 2016). Finally political trust is significantly lower for Indigenous people (−0.163) and about half of this effect is mediated via discrimination.

**Table 4:** KHB mediation analyses of extent to which discrimination mediates effect of nativity status on political trust.

			**Robust**	**Overall**
	**Estimate**		**SE**	**Mediation %**
**VS. CANADIAN-BORN WHITE**				
**Canadian-born person of color**				
Total effect	−0.033		0.047	
Direct effect	0.075		0.048	
Mediating effect	−0.108	^***^	0.021	329.64
**Foreign-born white**				
Total effect	0.191	^***^	0.022	
Direct effect	0.199	^***^	0.022	
Mediating effect	−0.008		0.018	−4.14
**Foreign-born person of color**				
Total effect	0.440	^***^	0.024	
Direct effect	0.521	^***^	0.015	
Mediating effect	−0.081	^***^	0.020	−18.43
**Indigenous**				
Total effect	−0.163	^***^	0.050	
Direct effect	−0.084	^*^	0.050	
Mediating effect	−0.079	^***^	0.019	48.54

* p < 0.05; ^**^p < 0.01;

*** *p < 0.001*.

Perceived discrimination mediates the ethnic gap in trust. We also sought to consider whether this relationship was reflective of the effects of race, nativity, and/or Indigeneity. Discrimination plays a greater mediating role between nativity and trust for immigrants who are also people of color. This difference occurs because people of color, irrespective of whether they were born in Canada or not, and Indigenous peoples report higher rates of discrimination than do either Canadian or foreign-born whites.

Immigrants have lower generalized trust and lower trust in specific others because of the discrimination they experience as racialized minorities rather than because they are immigrants *per se* (H3). In the case of generalized trust and trust in specific others the analysis of the 2013 Canadian General Social Survey shows that there is a mediating effect of discrimination on trust based on race but not immigrant status.

## Conclusion

Tensions with Muslims over the Burkini in France, support for the exit of the U.K. from the European Union, and even the recent debates about foreign home ownership in Canada clearly illustrate the challenges facing immigrant minorities in many countries. Underlying these challenges is a crisis of trust—a widening trust gap between immigrant and racialized minorities and majority populations in institutions and authorities.

But noting that there is a crisis of trust related to minority groups does not explain why it occurs. Minority group status is merely a container or “black box” for other experiences and characteristics (Tilly, 2001; Reskin, 2003). There is a need to identify the mechanism, that is, the process or set of experiences, through which these status group markers connect to trust. This paper contributes to this endeavor by considering the extent to which discrimination is the mechanism that might account for group differences in trust. Although it is widely believed that ethnic and racial gaps in trust stems from discrimination this argument has yet to be directly tested.

The reason for this gap is that, in the case of immigration, trust, and discrimination the focus has been on the universal effect of discrimination across immigrant groups, or for a smaller number of studies, on whether the effect of discrimination might matter more for some groups than for others. The fact that some groups—including immigrants—experience a lot more discrimination than others is left implicit. In order to take into account differential rates of discrimination, that is that some groups experience more discrimination than others, there needs to be a shift from a moderating approach to a mediating approach. This entails a shift from explaining overall aggregate levels of trust to explaining group-based *gaps* in trust. The limited number of studies in the trust literature that have attempted to explain group-based gaps in trust across ethnic and immigrant groups (e.g., see De Vroome et al., 2013; Hwang, 2017) have yet to consider the direct experience of discrimination or to use any kind of formal test of mediation.

The results clearly show that race needs to be disentangled from nativity status. This finding is important, especially in a context of huge changes in global migration patterns and increased migration of non-whites in both the European and North American contexts. In the case of generalized trust and trust in specific others the analysis of the 2013 Canadian General Social Survey shows that there is a mediating effect of discrimination on trust based on race and Indigeneity but not immigrant status. The results clearly show that both race and Indigeneity are important and that these need to be disentangled from nativity status. This finding is important, especially in a context of huge changes in global migration patterns and increased migration of non-whites in both the European and North American contexts.

If discrimination matters for the native-born this means that, irrespective of whether immigrant minority groups “catch up” in terms of other factors that affect trust, there is unlikely to be a catching up effect in terms of the trust of the second generation. This may in part explain why, even though Canada is generally a high trust country, it has not been immune to trust challenges: Black Lives Matter has resonated with the experiences of many in Canadian cities, Francophones consistently trust less, and there is an Inquiry into Murdered and Missing Indigenous women. None of the first two are recent immigrant groups and the third is Indigenous.

These results do, however, depend on the type of trust. In the case of political trust, the results help to explain previous work showing that members of some minority groups have higher political trust than majority group members. We find that individuals who are foreign-born show no difference in political trust or actually trust more than Canadian-born whites. This occurs because of competitive mediation, that is, a pattern where the mediated and direct effect are approximately the same size but operate in different directions (see Zhao et al., 2010). That the direct effect of being foreign-born is positive is likely because institutions in Canada are generally trustworthy—at least on a global scale and hence minority groups often look to the state for protection (Maxwell, 2010). However, this relationship does not appear to exist for those who have directly experienced discrimination. A further issue is that, in the case of political trust, for Indigenous peoples political it is lower and this is exacerbated by the direct experience of discrimination. All too often this group is omitted from the nativity-immigrant comparison, and it must be acknowledged that the distinction of place of birth may be irrelevant to many Indigenous peoples (e.g., see Deer, 2011; Fenelon, 2016).

Finally, there are a number of avenues for further research that emerge from the work presented here. First, the mediation approach used in this paper could be use either to explain gaps in other outcomes that vary between immigrants and the native-born. This might include economic outcomes such as income, political outcomes such as voting and social outcomes such as well-being and happiness. Second, the mediation approach could be extended to considering the role of other types of mediators including, demographics, socio-economic resources, and social capital. Although discrimination was often the most important factor this was not across the board and, in most instances it is partial mediation ranging from about 20–50%. Thus, about 50% in the gap in trust still requires explanation. Third, is that although we have focused on the direct experience of discrimination we do not wish to suggest that discrimination does not also matter because of its relationship to other trust correlates. Take, for example, education which is a form of human capital that leads to higher trust^10^. In the case of immigrants and racialized minority groups, in addition to overt discrimination, there are also specific discriminatory and colonial institutional histories that lead to lower general levels of the very factors such as education that in turn predict overall levels of integration and well-being.

## Author Contributions

All authors listed have made a substantial, direct and intellectual contribution to the work, and approved it for publication.

### Conflict of Interest Statement

The authors declare that the research was conducted in the absence of any commercial or financial relationships that could be construed as a potential conflict of interest.

## Footnotes

^1^In the Canadian context it is important to reflect on the use of the term “native-born.” The term native-born is used in a context where citizenship is jus soli or born on the soil. Inherent in the term native-born is a suggestion of originality. In a context of settler colonies such as Canada the original native-born are Indigenous people. The use of native-born to describe successive groups of Canadians suggests that the important difference in nativity is between groups of immigrants (whether they acquired their citizenship via jus soli or via naturalization) and the timing of their arrival rather than between Indigenous and non-Indigenous peoples. What this means is that the reference group in any comparison of Immigrant-native born should be Indigenous people rather than earlier immigrant groups such as the British and French. This is a project we are currently working on and we acknowledge this as a major limitation of the framework of this paper. We are grateful to Henry Yu for discussions with us on this point.

^2^The GSS file visible minority question asks whether the respondent is a visible minority or not, not their specific group identification. That said, the most recent census indicates that the vast majority of the visible minority population in Canada (61.3%) are South Asian, Chinese and Black (Statistics Canada, 2019). Also included in the visible minority group are “Filipinos, Latin Americans, Arabs, Southeast Asians, West Asians, Koreans, and Japanese.” (ibid).

^3^The presupposition in using the brthcan measure is that those born in Canada are not immigrants and that those born outside of Canada are immigrants. When this variable is used in combination with BPR_16 (landed immigrant status) the data show that while 8,164 are landed immigrants there are 726 individuals who are born outside of Canada who are not landed immigrants—e.g., most likely people born outside of Canada to Canadian parents. We re-ran all models omitting these individuals and the results are similar.

^4^See Appendix for an elaborated discussion of these approaches. Because they are only available for the foreign-born groups in our model, we do not include a number of measures such as years since arrival (yrarri), landed immigrant programs (LIP_10) and macro- geographic region of birth (brthmacr). Separate analyses (not shown but available from authors) show that, among the foreign-born population, people of color have a more recent average arrival date and are more likely to be refugees than white immigrants.

^5^The results of a regression of the discrimination variables on the immigration variables is provided in Table A1.

^6^This variable adjusts for age, sex, and region. Statistics Canada recommends using bootstrap weights. Because the KHB procedure does not currently allow for this, we conducted separate estimations of all models (without khb) using svyset [pweight = WGHT_PER], bsrweight(WTBS_001- WTBS_500) vce(bootstrap) dof(500) mse command (see http://statcan.gc.ca/pub/12-002-x/2014001/article/11901-eng.htm#a5). As should be expected the point estimates are similar and there are slight changes in the standard errors (see e.g., Kolenikov 2010). The substantive conclusion of the study do not change.

^7^It is worth noting that the logical presentation of the categories normalizes white Canadian as the “norm” from which all others deviate.

^8^Given the current and historical context of Indigenous-Canada relations (e.g., see Taiaiake, 1999; Ramos, 2006; Denis, 2015) the qualification should be made that the reported rates might have been higher for Indigenous people had a specific question been asked about discrimination related to being Indigenous. Further is that any such question might get at overt discrimination but not colonization.

^9^The percentage rate of missing data on each measure is as follows: general trust (2.5), specific trust (9.0), political trust (6.0), age (0.6), gender (0.6), marital status (0.8), rural (0.6), education (1.3), work status (0.8), volunteer (0.8), number of friends (2.1), political interest (1.1), ethnic discrimination (2.1), racial discrimination (2.2), discrimination in past 5 years (3.9), ethno-racial/Indigenous origin (1.0). Since this rate of missingness overall is relatively low and because specific and political trust are dependent variables we do not use multiple imputation (see e.g., Von Hippel, 2007).

^10^The lower rates of education for Indigenous people in the Canadian context are, for example, themselves a legacy of more than a century of discrimination and genocide via the Indian Act and concomitant forced attendance at Indian Residential Schools.

## Appendix

### Brief Overview of Ses, Social Capital, and Demographic Approaches

One line of argumentation is that variability in SES factors such as income and education across ethnic groups may be a factor in explaining trust differences across immigrant and native-born groups (Soroka et al., 2006). The experience of being one of the societal “have” groups means better treatment and hence, more social trust (Putnam, 2001; Delhey and Newton, 2005; Rothstein and Uslaner, 2005). That is, individuals with higher socio-economic status are more likely to benefit from existing social and political institutions and hence to view them more favorably (Newton, 2001; Uslaner and Brown, 2005; Wu and Wilkes, 2017). As a result, their trust in such organizations is higher. De Vroome's et al. (2013) study of the difference in social and political trust between native Dutch respondents and first and second generation Moroccan and Turkish immigrants shows that a significant proportion of the trust gap can be attributed to the lower socio-economic status of immigrant groups. In contrast, Zerfu et al's (2008) study of ethnicity and trust in eight African countries finds that class variables do not explain the effect of ethnicity on trust (see p 167).

A second line of argumentation is that the gap in trust may stem from group differences in social capital. Social capital (Putnam, 1993, 1995) refers to “features of social organization such as networks, norms, and social trust that facilitate coordination and cooperation for mutual benefit.” (1995:66). Putnam argues that “civic engagement and social connectedness” are especially important for the creation of social capital (ibid). Although there is some debate in the broader literature as to whether trust is part of, or an outcome of social capital, and well as whether social trust is a form of social capital that leads to institutional trust (Catterberg and Moreno, 2006), the general argument is that “a dense network of voluntary associations and citizens organizations help to sustain civil society and community relations in a way that generates trust and cooperation between citizens” (Newton, 2001). The De Vroome et al. (2013) study shows that social capital measures, such belonging to associations and feeling integrated in society account for some of the difference in trust between native Dutch respondents and first and second generation Moroccan and Turkish immigrants. Still, Maxwell (2010) considered whether the difference in political trust between Muslims and Christians in Britain was due to the fact that political trust is higher among the former group as a result of differing degrees of political efficacy. He found that while political efficacy does increase political trust for each group, the mean levels across these groups are very similar (ibid).

Finally, there has been some suggestion that the gap in trust may be the result of group demographic differences. Age has been shown to increase trust because older individuals, particularly those from a long “civic” generation are most likely to be civically and politically engaged and hence to trust more (Putnam, 1993; see also Uslaner, 2011). Marital status has also been found to correlate to trust either because the kind of people who get married also tend to have other kinds of social capital related to trust or because marriage itself increases trust in others (Helliwell and Putnam, 2004). Gender has also been found to correlate with both social and political trust. Women tend to have lower trust in generalized others (Mewes, 2014) but their political trust tends to be higher (Mishler and Rose, 2001; except see Catterberg and Moreno, 2006). While Soroka et al. (2006) consider the extent to which age, immigrant status and religion can explain differences in generalized and strategic trust between British/Northern European and Aboriginal, Quebec Francophones, Southern Europeans and Eastern Europeans.

**Appendix 1:** Regression of ethno-racial categories on discrimination (with bootstrapped standard errors).

	**Ethnic discrimination**	**Racial discrimination**	**Any discrimination**
**VS. CANADIAN-BORN WHITE**			
Canadian-born person of color	2.302^***^	2.519^***^	1.033^***^
	–17.42	–19.11	–8.9
Foreign-born white	0.668^***^	–0.106	–0.0445
	–7.3	(–0.80)	(–0.76)
Foreign-born person of color	1.982^***^	2.116^***^	0.632^***^
	–27.34	–28.39	–11.65
Aboriginal/Indigenous	1.659^***^	1.622^***^	0.865^***^
	–14.12	–13.11	–8.94
Intercept	–2.868^***^	–3.040^***^	–0.972^***^
	(–59.00)	(–57.76)	(–41.08)
*N*	27,032	27,019	26,545

*t statistics in parentheses*.

*^*^p < 0.05, ^**^p < 0.01*,

*** p < 0.001
